# Production, characterization and antioxidant analysis on the *Undaria*-based alcoholic beverages using response surface method and HS-SPME-GC × GC-TOF-MS

**DOI:** 10.1016/j.fochx.2025.102428

**Published:** 2025-04-01

**Authors:** Fangru Nan, Xinyi Li, Jia Feng, Junping Lv, Qi Liu, Xudong Liu, Yang Liu, Ruikai Zhang, Baoqing Bai, Shulian Xie

**Affiliations:** a School of Life Science, Shanxi University, Taiyuan 030006, China; b Shanxi Key Laboratory for Research and Development of Regional Plants, Shanxi University, Taiyuan 030006, China; c Xinghuacun College of Shanxi University (Shanxi Institute of Brewing Technology and Industry), Taiyuan 030006, China

**Keywords:** *Undaria pinnatifida*, Volatile compounds, Response surface methodology, Polysaccharides

## Abstract

*Undaria pinnatifida*, a nutrient-rich seaweed, holds potential for the alcoholic beverage industry. This study optimized the ultrasonic processing of *Undaria* blend liquor (UBL) and the fermentation of *Undaria* fermented wine (UFW) while identifying volatile components and assessing antioxidant properties. After optimization, UBL had a polysaccharide content (PC) of 0.66 g/L and an alcohol content (AC) of 39.2 % vol, while UFW showed a PC of 9.81 g/L and an AC of 8.3 % vol. HS-SPME-GC × GC-TOF-MS analysis identified 34 characteristic volatile compounds, with esters as the predominant class. UBL was featured by notably high levels of ester compounds, while UFW contained fatty acids leading to distinct flavor profiles. Antioxidant assays revealed that both beverages demonstrated free radical scavenging activity in a dose-dependent manner. These findings highlight the potential of *Undaria* as a novel resource for developing functional and flavorful alcoholic beverages, contributing to innovation in the food and liquor industries.

## Highlights

• Using *Undaria* as raw material, two different brewing processes were explored.
• The aroma components were analyzed by non-targeted metabolomics.
• 34 key aroma compounds were identified in *Undaria* blended liquor and fermented wine.
• *Undaria* blended liquor and fermented wine have certain antioxidant capacity.

## 1 Introduction

*Undaria pinnatifida*, a type of common brown algae, is rich in protein, fat, crude fiber and a variety of bioactive substances, such as vitamins, amino acids, trehalose and alginate (Hou et al., 2024; Lee & Kim, 2024). A number of studies have shown that *Undaria* has antioxidant, anti-inflammatory, anti-tumor, anti-hypertensive, anti-viral and anti-obesity properties (Han et al., 2002; Khan et al., 2007; Lee et al., 2004; Maeda et al., 2009; Mohamed, 2014; Vishchuk et al., 2011). The fucoidan in *Undaria* has great potential as a functional food to reduce disease or as a supplement to alternative therapies (Zhao et al., 2018). *Undaria* is one of the popular edible seaweeds in Asia, particularly in China, Japan and South Korea (Wang et al., 2018). *Undaria* is usually added to noodles, soups, salads or pickled foods as food excipients (Yoshinaga et al., 2018). Prabhasankar et al. (2009) added wakame (U. *pinnatifida*) to pasta, which enhanced the interaction between starch granules and the protein matrix by 20 %. Sun et al. (2019) showed that the addition of polysaccharide extract from the sporophyll of *Undaria* could increase the antioxidant activity of biscuits and delay lipid oxidation, thus effectively prolonging the shelf life of biscuits.

Alcoholic beverages have been a significant part of human culture for millennia, reflecting changes in societal preferences, technological advancements, and economic developments (Dietler, 2006; Mandelbaum, 1965). Recently, heightened public interest in health and chronic disease prevention has drawn attention to drinking as a major modifiable factor for many chronic conditions (Zhou et al., 2016). Studies indicate that the health benefits of moderate alcohol consumption are largely attributable to the bioactive components present in alcoholic beverages (Lin et al., 2023). Fermentation is the key process in alcoholic beverage production and an innovative choice for new seaweed products (Francezon et al., 2021) (Stanzer et al., 2023). Through fermentation, it can not only induce the release of certain organic substances (such as amino acids), change the flavor of algae, but also further transform these substances to produce new flavor active compounds (Chen et al., 2024). Ultrasonics is a new type of food processing technology, which is widely used in various brewing processes, such as extraction, fermentation, aging and sterilization (Liu et al., 2024). The study of Xue et al. (2021) showed that compared with the other three conventional extraction methods (hot water, acidified ethanol and enzyme-assisted extraction), the ultrasonic-assisted enzymatic method had the highest anthocyanin yield and extraction efficiency in raspberry wine residue. The research of Hao et al. (2023) showed that the use of ultrasonic technology can simplify the brewing process of Chinese rice wine, shorten the brewing time and improve the volatile flavor.

The purpose of this study was to optimize the ultrasonic process of *Undaria* blend liquor (UBL) and the fermentation process of *Undaria* fermented wine (UFW), identify volatile components, and assess antioxidant properties. The research results will provide scientific basis for the development of *Undaria* liquor and wine industry.

## 2 Material and methods

### 2.1 Materials and strains

*Undaria* was sourced from October Paddy Food Group Co., Ltd. The base liquor (42 %, 53 %, and 65 % vol Daqu Fen-flavor Baijiu) was obtained from Shanxi Hongdequan Wine Co., Ltd., producers of a traditional Chinese distilled alcoholic beverage. *Saccharomyces cerevisiae* 2.35 and *Lactobacillus acidophilus* 1.208 were obtained from the Guangdong Provincial Microbial Strain Conservation Center (GDMCC). The yeast strain was cultured in YPED medium (1 % [*w*/*v*] yeast extract, 2 % [w/v] peptone, and 2 % [w/v] glucose) at 28 °C (Yan et al., 2023). The lactic acid bacteria were cultured in MRS Broth medium (Guangdong Huankai Microbial Technology Co., Ltd., Guangdong, China) at 37 °C (Taillandier et al., 1996).

### 2.2 Optimization of *Undaria* blended liquor (UBL)

2 g *Undaria* powder was soaked in 98 mL base liquor (BL), stirring thoroughly and processed using an ultrasonic crushing instrument (SCIENTZ-950E, Ningbo Scientz Biotechnology Co., Ltd., Ningbo, China). After centrifuged at 5000 rpm for 10 min, a clear liquid was obtained. The UBL optimization process was adapted from Gong et al. (2022) with minor modifications. Single-factor experiments were conducted to determine the initial values of the variables. The Box-Behnken design (BBD) of response surface methodology (RSM) was used to optimize the ultrasonic conditions across three factors (A: base liquor degree, B: ultrasonic time, and C: ultrasonic power) at three levels (Table 1). The response surface analysis involved 17 experimental runs, each repeated three times (Table 2). The optimized ultrasonic parameters were obtained through a regression equation, with PC (Y1) as the response value, measured using the 3,5-dinitrosalicylic acid method (Hu et al., 2008). Total phenol (TP) was measured using the Folin-Ciocalteu method (Santos-Zea et al., 2011).

**Table 1:** Coded levels of independent variables and response values of Box-Behnken design (BBD) for UBL.

Factor	Name		Level	
		-1	0	1
A	Wine base degree (%vol)	42	53	65
B	Ultrasonic time (min)	40	50	60
C	Ultrasonic power (W)	80	95	110
Response Value	Name	Minimum	Maximum	Average
Y1	PC (g/L)	0.65	0.67	0.66

**Table 2:** Box-Behnken design (BBD) matrix and response for UBL.

Trial No.	A	B	C	PC (Y1)
1	65	50	80	0.44
2	53	50	95	0.50
3	42	40	95	0.60
4	42	60	95	0.61
5	53	50	95	0.50
6	53	40	110	0.50
7	53	50	95	0.52
8	65	40	95	0.41
9	53	40	80	0.49
10	65	60	95	0.43
11	42	50	110	0.64
12	53	60	110	0.48
13	42	50	80	0.62
14	53	60	80	0.49
15	53	50	95	0.51
16	65	50	110	0.45
17	53	50	95	0.51

Note: A Wine base degree (%vol), B Ultrasonic time (min), C Ultrasonic power (W), PC (g/L).

### 2.3 Optimization of Undaria fermentation wine (UFW)

A sucrose aqueous solution was prepared by dissolving 220 g of sucrose in distilled water, adjusting the pH to 5.0 and autoclaving at 110 °C for 15 min. Each fermentation experiment was executed under 19°Brix of soluble solids and in a 100 mL volume including 85 mL sucrose aqueous solution, 2 g autoclaved *Undaria* powder and 6 % mixed bacterial solution (the inoculation ratio of *S. cerevisiae* to L. *acidophilus* was 4:2) and distilled water. The fermentation was carried out with a cell concentration of 1 × 10^7^ CFU/mL for both *S. cerevisiae* and L. *acidophilus*. The UFW optimization process followed the method of Li et al. (2024), with minor modifications. Single-factor experiments were conducted to determine the initial values of the variables. The BBD was employed to optimize fermentation conditions across three factors (A: fermentation time, B: inoculation amount, and C: fermentation temperature) at three levels (Table 3). The response surface analysis involved 17 runs, each repeated three times (Table 4). The optimized fermentation parameters were obtained by the regression equation. The PC (Y2) is the response value and determined by subtracting the content of reducing sugar determined by the 3,5-dinitrosalicylic acid method (Hu et al., 2008) from the total sugar content determined by the phenol‑sulfuric acid method (Zhai et al., 2018). TP measurement followed the method mentioned previously. Total soluble solids (TSS) were measured using a hand-held refractometer (PAL-1, ATAGO CHINA Guangzhou Co., Ltd., Guangdong, China), and pH was assessed using a pH meter (a-AB33PH ZH, Ohaus Instruments Co., Ltd., Jiangsu, China). AC was determined according to the Chinese national standard GB 5009.225–2016 using a rotary evaporator (RE52CS, Shanghai Yarong Biochemical Instrument Factory, Shanghai, China), and determined by an alcohol meter (Huaou Instrument and Meter Factory, Hebei, China).

**Table 3:** Coded levels of independent variables and response values of Box-Behnken design (BBD) for UFW.

Factor	Name		Level	
		-1	0	1
A	Fermentation temperature (°C)	19	22	25
B	Inoculation amount (%)	4	6	8
C	Fermentation time (d)	9	11	13
Response Value	Name	Minimum	Maximum	Average
Y1	PC (g/L)	9.76	9.88	9.81

**Table 4:** Box-Behnken design (BBD) matrix and response for UFW.

Trial No.	A	B	C	PC (Y2)
1	22	6	11	10.16
2	22	8	13	7.72
3	22	6	11	9.91
4	19	6	9	7.22
5	19	4	11	6.02
6	19	8	11	6.80
7	22	6	11	10.09
8	22	6	11	9.23
9	25	6	9	6.57
10	22	4	9	5.26
11	22	8	9	7.30
12	25	4	11	5.54
13	22	4	13	6.99
14	25	8	11	6.28
15	19	6	13	8.42
16	25	6	13	8.76
17	22	6	11	9.81

Note: A Fermentation temperature (°C), B Inoculation amount (%), C Fermentation time (d), PC (g/L).

### 2.4 Determination of antioxidant activity in vitro

#### 2.4.1 ABTS scavenging activity

The ABTS radical scavenging assay was adapted from Re et al. (1999). Samples were mixed with the ABTS solution in a 96-well plate and incubated in the dark at room temperature for 30 min. Absorbance was measured at 734 nm. Vitamin C (Vc) was used as the positive control. The scavenging rate was calculated using the absorbance values of the sample-ABTS mixture (A_1_), sample-ethanol mixture (A_2_), and control (A_0_). The scavenging rate was calculated by the following formula:

(2) $$\text{ABTS scavenging rate}\;\left(\%\right)=1-\left(A_{1}-A_{2}\right)/A_{0}\times 100\%$$

#### 2.4.2 DPPH scavenging activity

The DPPH free radical scavenging assay was adapted from Amarowicz et al. (2000) with minor adjustments. Samples were mixed with DPPH solution in a 96-well plate, incubated at room temperature for 30 min, absorbance was measured at 517 nm. Vc was used as the positive control. The scavenging rate was calculated using the absorbance values of the sample-DPPH mixture (A_1_), sample-ethanol mixture (A_2_), and control, ethanol-DPPH (A_0_). The scavenging rate was calculated using the following formula:

(3) $$\text{DPPH scavenging rate}\;\left(\%\right)=1-\left(A_{1}-A_{2}\right)/A_{0}\times 100\%$$

#### 2.4.3 ·OH scavenging activity

The hydroxyl radical (·OH) scavenging assay was assessed following the method of Smirnoff and Cumbes (1989) with minor adjustments. Samples were mixed with ferrous sulfate and salicylic acid-ethanol solution, and the reaction was initiated with hydrogen peroxide, incubated at 37 °C for 30 min and absorbance was measured at 510 nm. Vc served as the positive control. The absorbance of the sample‑hydrogen peroxide mixture was recorded as A_1_. The absorbance of the sample without hydrogen peroxide was noted as A_2_. The scavenging rate was calculated using the following formula:

(4) $$\cdot \mathrm{OH}\;\text{scavenging rate}\;\left(\%\right)=1-\left(A_{1}-A_{2}\right)/A_{0}\times 100\%$$

#### 2.4.4 Total reducing power determination

The reducing power of the samples was evaluated using the Oyaizu method (Oyaizu, 1988) with slight modifications. The sample solution was mixed with phosphate buffer and potassium ferricyanide, incubated at 50 °C for 20 min, cooled, and mixed with distilled water and ferric chloride. Absorbance was measured at 700 nm. Vc was used as the positive control.

### 2.5 Volatile composition analysis (HS-SPME-GC × GC-TOF-MS)

Following the method by Shen et al. (2023), our method was modified slightly. Samples of 6 mL were weighed in a 20 mL headspace vial with 1.6 g of NaCl. A 10 μL internal standard (20 mg/L 2-octanol in anhydrous ethanol solution) was added to the headspace vial and vortexed for 60 s. The headspace solid phase microextraction multifunctional automatic sampling system was provided by Guangzhou Zhida Laboratory Technology Co., Ltd. Volatile compounds were adsorbed by SPME Arrow fibers (CAR/PDMS, pHase thickness 120 μm, outer diameter 1.1 mm). The headspace vial was pre-incubated at 60 °C for 30 min, extracted at 60 °C for 30 min, the extraction depth was 30 mm, the injection depth was 65 mm, and the desorption time was 30 s. The GGT 0620 two-dimensional gas chromatography time-of-flight mass spectrometer (Guangzhou Hexin Instrument Co., Ltd.) was equipped with a DB-WAX main column (30 m × 0.25 mm × 0.25 μm) and a DB − 17 auxiliary column (1.2 m × 0.18 mm × 0.18 μm) for analysis. The temperature of the forward sample port was 240 °C, and there was no shunt during injection. The carrier gas was helium, and the flow rate was 1 mL/min. The initial temperature of 40 °C was maintained for 2 min, then increased to 230 °C at a rate of 5 °C/min for 7 min. A high-pressure modulation column (1.2 m × 0.25 mm) was used, with a modulation period was 1 s/5 s. The interface temperature was set at 240 °C, and the ion source temperature was set at 230 °C. The voltage of the mass spectrometry detector was −1480 V, the ionization energy was 70 eV, the mass range was 30–500 amu, the data acquisition rate was 101 spectra/s, and the solvent delay was 3 min.

Canvas software was used for 2D data processing and to generate a full 2D TIC contour map. Peaks with a signal-to-noise ratio greater than 10 were identified, with each peak representing a compound. Each compound is characterized by a pair of retention times: the first-dimensional retention time (min) along the x-axis and the second-dimensional retention time (s) along the y-axis. Volatile compounds were identified by comparing their one-dimensional and two-dimensional GC × GC-TOF-MS retention times and mass spectra with those in the NIST 17 database. The compound identity was confirmed based on the retention time, retention index (RI), and spectral matching value of >700 with the NIST library. The concentration of volatile compounds was calculated by dividing the peak area of each compound by the peak area of the internal standard.

### 2.6 Data processing and multivariate analysis

All the results were expressed as mean values ± standard deviation (SD).The one-factor analysis of variance (ANOVA) and Duncan test was performed using the SPSS 27 software (IBM SPSS Inc., Armonk, NY, USA). Significance was established at *p* < 0.05. The optimization was conducted by response surface methodology with Design Expert 12 (Stat Soft Inc., Minneapolis, MN, USA). Multiple regression and analysis of variance (ANOVA) were used to analyze the data to fit the quadratic polynomial model. Statistical significance was assumed when *p* < 0.05. Principal component analysis (PCA), partial least squares discriminant analysis (PLS-DA) and orthogonal partial least-squares discrimination analysis (OPLS-DA) were conducted using SIMCA 14.1 software (Umetrics, Umea, SWE). The heatmap, and volatile compounds analysis plot were conducted using the metabolomic data analysis tool, ChiPlot (https://www.chiplot.online).The rest of the drawing is done by origin 2021 (Origin Lab Corporation, Northampton, USA).

## 3 Results

### 3.1 Preparation of UBL

#### 3.1.1 The effects of base liquor degree, ultrasonic time, and ultrasonic power on PC

**Fig. 1:**
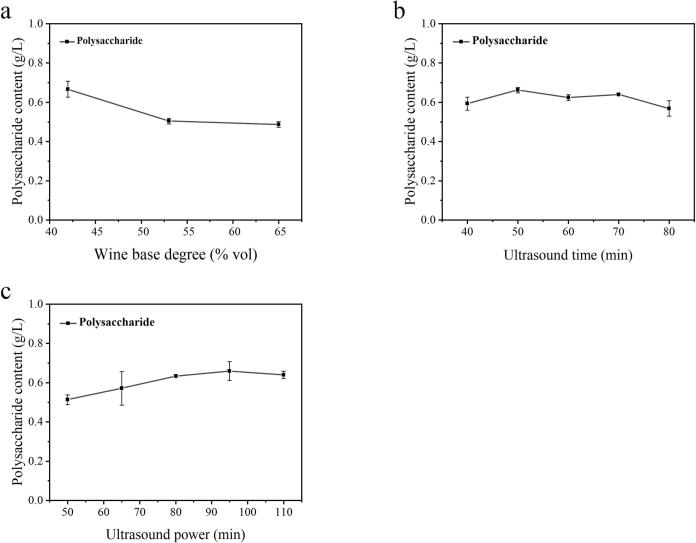
A single-factor test of UBL. The effects of wine base degree (a), ultrasonic time (b) and ultrasonic power (c) on the PC in UBL were analyzed with PC as the main index.

Fig. 1(a-c) shows the effects of three single factors-base degree, ultrasonic time, and ultrasonic power—on the PC of UBL, with the other two factors held constant. The highest values were observed at the base liquor degree of 42 % vol, ultrasonic time of 50 min and ultrasonic power of 95 W, respectively.

#### 3.1.2 Optimization of ultrasonic conditions

The regression model of UBL parameters obtained from BBD experiments was as follows:

(6) $$\begin{array}{l} Y=0.5029-0.0922A+0.0014B+0.0032C+0.0005\mathrm{AB}-0.0029\mathrm{AC}\\ -0.0074\mathrm{BC}+0.0316A^{2}-0.0222B^{2}+0.0040C^{2} \end{array}$$

As shown in Table 5, the *p*-value is used to evaluate the significance of the model. Higher *F*-value (86.08) and lower probability value (*p* < 0.0001) indicated that the model was significant. The higher R^2^ value (0.9910) indicates that the good fit of the formula regression model is higher. The adjusted R^2^ value (0.9795) indicates that the model explains 97.95 % of the variability in the response, reflecting its accuracy and robustness.

**Table 5:** ANOVA evaluation of linear, interaction and quadratic terms of response variables and model predicted coefficients for PC of UBL.

Source	SS	DF	MS	*F*-value	*p*-value	Significance
Model	0.0734	9	0.0082	86.08	<0.0001	**
A	0.0680	1	0.0680	717.42	<0.0001	**
B	0.0000	1	0.0000	0.1585	0.7024	
C	0.0001	1	0.0001	0.8410	0.3896	
AB	8.550E-07	1	8.550E-07	0.0090	0.9270	
AC	0.0000	1	0.0000	0.3673	0.5636	
BC	0.0002	1	0.0002	2.34	0.1700	
A^2^	0.0042	1	0.0042	44.11	0.0003	**
B^2^	0.0021	1	0.0021	21.80	0.0023	**
C^2^	0.0001	1	0.0001	0.7167	0.4252	
Residual	0.0007	7	0.0001			
Lack of fit	0.0005	3	0.0002	4.03	0.1057	
Pure error	0.0002	4	0.0000			
total	0.0741	16				
R^2^		0.9910				
R_ad_j^2^		0.9795				

Note: A, B and C represent the base degree of wine, ultrasonic time and ultrasonic power respectively; SS, DF and MS represent square sum, degree of freedom and mean square respectively. * was significant difference (*P* < 0.05), * * was extremely significant difference (*P* < 0.01).

**Fig. 2:**
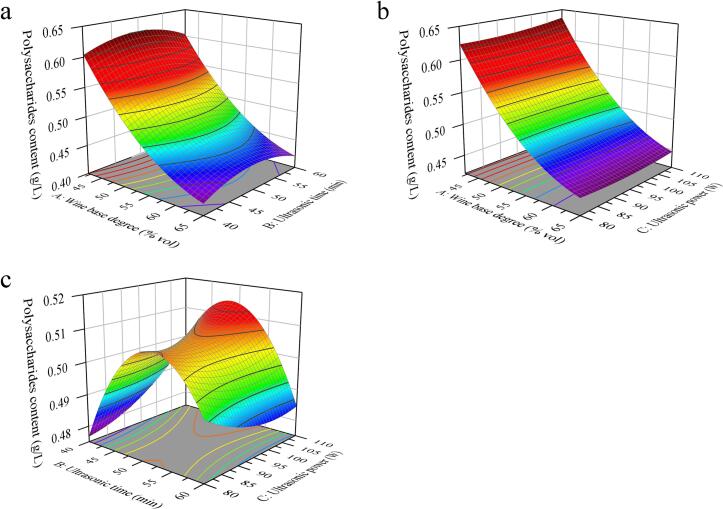
3D response surface plots of UBL. (a) Effect of the wine base degree and ultrasonic time. (b) Effect of the wine base degree and ultrasonic power. (c) Effect of the ultrasonic time and ultrasonic power.

The 3D response surface curve of the influence of various factors on the PC of UBL are presented in Fig. 2, illustrating the interactions among factors in the ultrasonic process. Fig. 2a shows the effect of the base liquor degree (A), ultrasonic time (B), and their interaction on the PC when the ultrasonic power (C) is fixed at 95 W. The results showed that PC decreased with the increasing base liquor degree and ultrasonic time. As shown in Fig. 2b, the PC decreased with the increase of base liquor degree (A) and ultrasonic power (C). As shown in Fig. 2c, the effects of ultrasonic time (B), ultrasonic power (C) and their interactions on PC were relatively minor.

The optimal process parameters were: base liquor degree 42 % vol, ultrasonic time 48.532 min, and ultrasonic power 110 W, with the highest predicted PC reaching 0.637 g/L. The results were verified under the experimental conditions of base liquor degree 42 % vol, ultrasonic time 49 min and ultrasonic power 110 W. The average PC of UBL was 0.66 g/L, close to the predicted value. This consistency validates the accuracy and reliability of the process conditions optimized by RSM, affirming the suitability of the model. The AC under optimal conditions was 39.2 % vol.

### 3.2 Preparation of UFW

#### 3.2.1 The effects of fermentation temperature, inoculum amount, and fermentation time on PC

**Fig. 3:**
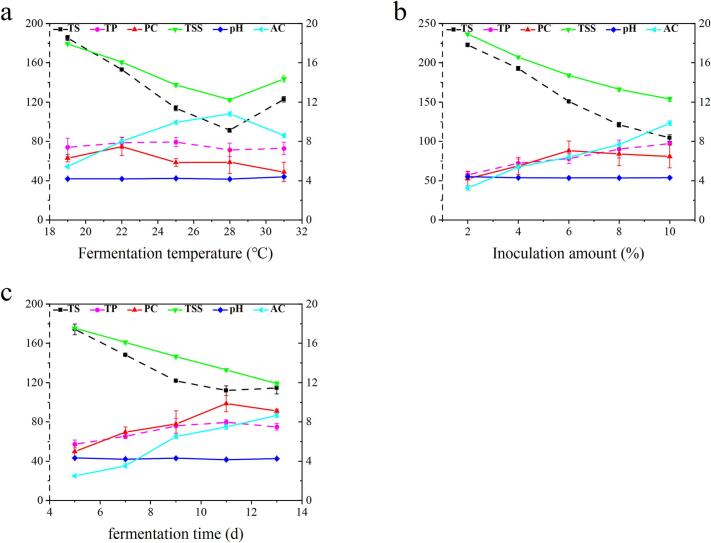
Single factor test of UFW. Note: Total sugar (TS), total phenol (TP), polysaccharide (PC), total soluble solids (TSS), alcohol content (AC). TS (g/L) and TP (mg/L) were ordinated to the left Y axis, while PC (g/L), TSS (°Brix), pH and AC (% vol) were ordinated to the right Y axis. The effects of fermentation temperature (a), inoculation amount (b) and fermentation time (c) on the content of polysaccharide in UFW were analyzed with PC as the main index.

Fig. 3(a-c) shows the effects of three factors—fermentation temperature, inoculum amount, and fermentation time—on the PC of UFW, with the other two factors held constant. The highest values were observed at fermentation temperature of 22 °C, inoculum amount of 6 % and fermentation time of 11 days, respectively.

The effects of fermentation temperature, inoculum amount, and fermentation time on TS, TP, TSS, pH, and AC were in Fig. 3. As fermentation temperature, inoculum amount, and fermentation time increased, AC and TP gradually increased, while TS and TSS decreased, and pH remained relatively unchanged.

#### 3.2.2 Optimization of fermentation conditions

The regression equations for PC (Y2) with fermentation temperature (A), inoculation amount (B), and fermentation time (C) are as follows.

(7) $$\begin{array}{l} Y=9.84-0.1643A+0.5356B+0.6945C-0.0100\mathrm{AB}+0.2472\mathrm{AC}\\ -0.3252\mathrm{BC}-1.38A^{2}-2.30B^{2}-0.7168C^{2} \end{array}$$

As shown in Table 6, the *p*-value was used to evaluate the significance of the model. The higher *F*-value (33.24) and lower probability value (*p* < 0.0001) indicated that the model was significant. The higher R^2^ value (0.9771) indicates a good fit of the regression model. The adjusted R^2^ value (0.9477) indicates that the model explains 94.77 % of the variability in the response, reflecting its accuracy and robustness.

**Table 6:** ANOVA evaluation of linear, interaction and quadratic terms of response variables and model predicted coefficients for PC of UFW.

Source	SS	DF	MS	*F*-value	*p*-value	Significance
Model	42.50	9	4.72	33.24	<0.0001	**
A	0.2159	1	0.2159	1.52	0.2575	
B	2.29	1	2.29	16.15	0.0051	**
C	3.86	1	3.86	27.16	0.0012	**
AB	0.0004	1	0.0004	0.0028	0.9592	
AC	0.2444	1	0.2444	1.72	0.2311	
BC	0.4231	1	0.4231	2.98	0.1280	
A^2^	8.01	1	8.01	56.40	0.0001	**
B^2^	22.35	1	22.35	157.35	0.0001	**
C^2^	2.16	1	2.16	15.23	0.0059	**
Residual	0.9945	7	0.1421			
Lack of fit	0.4491	3	0.1497	1.10	0.4469	
Pure error	0.5454	4	0.1364			
total	43.49	16				
R^2^		0.9771				
R_ad_j^2^		0.9477				

Note: A, B and C represent the base degree of wine, ultrasonic time and ultrasonic power respectively; SS, DF and MS represent square sum, degree of freedom and mean square respectively. * was significant difference (*P* < 0.05), ** was extremely significant difference (*P* < 0.01).

**Fig. 4:**
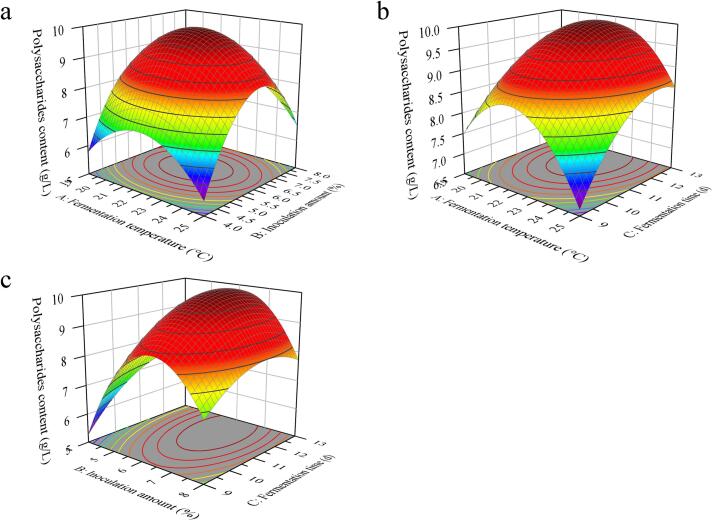
3D response surface plots of UFW. (a) Effect of the fermentation temperature and inoculation amount. (b) Effect of the fermentation temperature and fermentation time. (c) Effect of the inoculation amount and fermentation time.

The 3D response surface curves showing the influence of various factors on the PC of UFW are presented in Fig. 4, illustrating the interactions among factors in the fermentation process. The shape of the contour plot reflects the strength of the interactions between factors. From the contour plots, the shapes of the interactions AC, BC, and AB are oval, indicating that the interactions between any two of the fermentation temperature (A), inoculation amount (B), and fermentation time (C) have complex effects on the fermentation system.

The optimum conditions were: fermentation temperature 21.925 °C, inoculation amount 6.167 %, and fermentation time 11.925 days, with the highest predicted PC being 10.024 g/L. Considering the actual process, the parameters were adjusted to a fermentation temperature set of 22 °C, an inoculation amount of 6.2 %, and a fermentation time of 12 days. Three parallel experiments were conducted under these adjusted conditions, yielding a PC of 9.81 g/L, indicating that the measured value was very close to the predicted value. This confirms the accuracy and reliability of the response surface optimization process conditions, and verifies the applicability of the model. The AC under optimal conditions was 8.3 % vol.

### 3.3 Analysis of volatile components and multivariate statistical

**Fig. 5:**
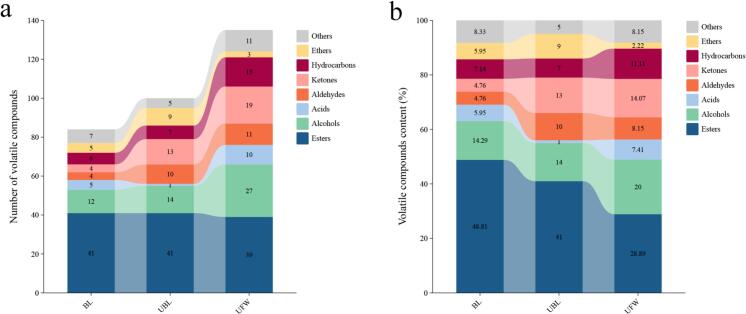
Volatile compounds analysis in BL, UBL and UFW. (a) number of volatile compounds. (b) volatile compounds content.

A total of 215 metabolites were identified by analyzing the volatile compounds in the BL, UBL, and UFW (Supplementary Table S1). These included 77 esters, 36 alcohols, 11 acids, 16 aldehydes, 25 ketones, 23 hydrocarbons, 12 ethers, and 15 other compounds. The analysis of the number and content of volatile compounds in BL, UBL, and UFW is shown in Fig. 5. It revealed that ester content was highest in BL, UBL, and UFW. Alcohols and ketones followed. Compared with BL and UBL, UFW contained the highest number of volatile compounds.

**Fig. 6:**
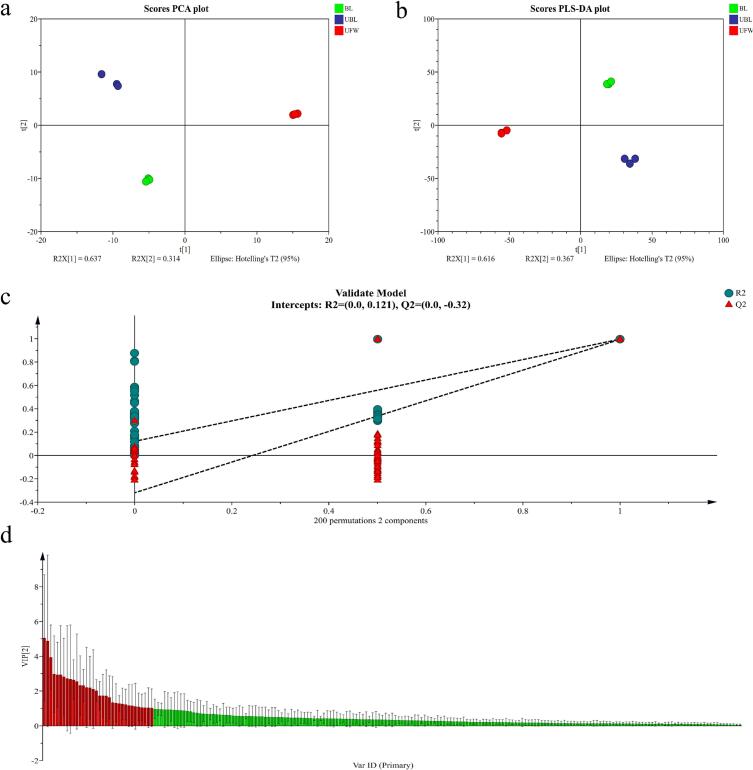
Multivariate statistical analysis of BL, UBL and UFW: (a) PCA score plot; (b) PLS-DA score plot; (c) Cross-validation plot of the PLS-DA model with 200 permutations; (d) VIP plot.

The data obtained from HS-SPME-GC × GC-TOFMS detection are multidimensional. To better understand the metabolic patterns of BL, UBL, and UFW, multivariate statistical analysis was employed to explore the differences in metabolites. First, as shown in Fig. 6a, PCA revealed that the first two principal components accounted for 63.7 % and 31.4 % of the variance, respectively. This analysis effectively distinguished BL, UBL, and UFW along the PC1 axis. Subsequently, PLS-DA was applied to further investigate the metabolites that distinguish these three samples. The PLS-DA model was robust, with R^2^X = 0.982, R^2^Y = 0.997, and Q^2^ = 0.994 (Fig. 6b). Cross-validation confirmed the reliability of the model (R^2^ = 0.121, Q^2^ = −0.32), indicating significant differences in the metabolic profiles among the samples (Fig. 6c). The red part of the VIP plot represents metabolites with VIP > 1 (Fig. 6d).

**Fig. 7:**
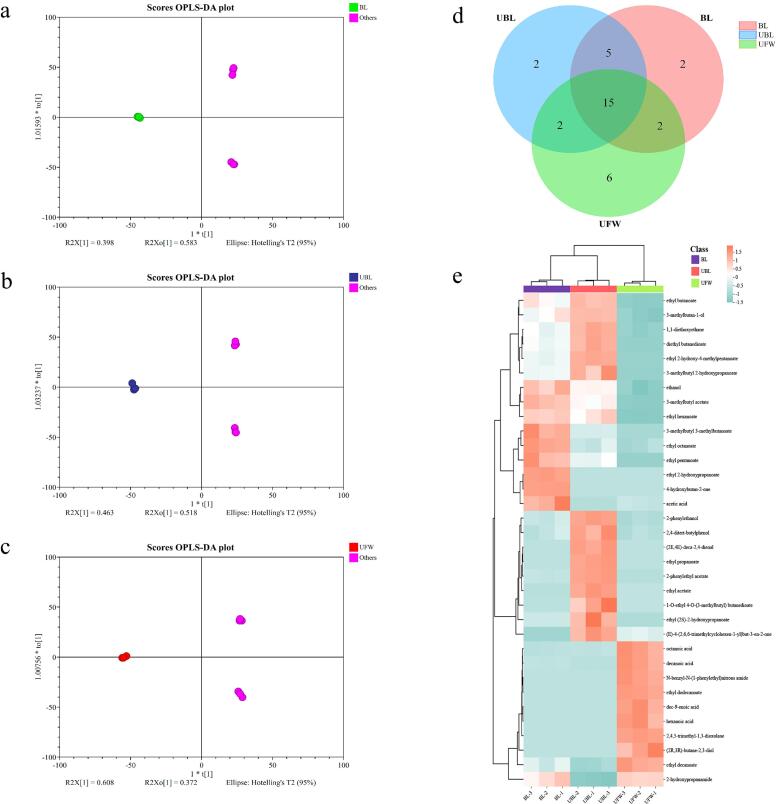
Validation of characteristic volatile compound for BL, UBL and UFW. OPLS-DA score plots of LB (a), UBL (b) and UFW (c) compared to the other two groups. (d) Venn plot. (e) Heatmap and hierarchical cluster analysis (HCA) of signature compounds in BL, UBL and UFW.

To further differentiate sample groups, OPLS-DA was utilized. This analysis successfully separated BL, UBL, and UFW from each other (Fig. 7a-c). The OPLS-DA model demonstrated strong interpretability and fit, with R^2^ values of 0.989, 0.987, and 0.988, and Q^2^ values of 0.997, 0.996, and 0.996, respectively. Furthermore, Fold-Change (FC) analysis was employed to compare the absolute value of changes between the two groups. Based on ANOVA (*p* ≤ 0.05) and FC (≥ 2 or ≤ 0.5), the model identified 34 significantly different metabolites (Table 7). Among these, ester compounds were the most prevalent. There were also significant differences in the types and concentrations of esters among BL, UBL and UFW (*p* < 0.05). The number of esters in UFW (10 esters) was higher than in BL (8 esters) and UBL (9 esters). The main esters detected were ethyl octanoate, diethyl butanedioate, ethyl hexanoate, 3-methylbutyl acetate, and ethyl pentanoate. The Venn diagram shows that there are 15 co-owned compounds among BL, UBL, and UFW (Fig. 7d). Two compounds are unique to BL: 4-hydroxybutan-2-one and ethyl 2-hydroxypropanoate. Two compounds were unique to UBL: ethyl propanoate and (2E,4E)-deca-2,4-dienal. Six compounds were unique to UFW: ethyl dodecanoate, (2*R*,3*R*)-butane-2,3-diol, dec-9-enoic acid, hexanoic acid, 2,4,5-trimethyl-1,3-dioxolane, and N-benzyl-N-(1-phenylethyl)nitrous amides. To better understand the differences in the content of these volatile compounds, heatmap and hierarchical cluster analysis (HCA) were employed for clustering and visual analysis, showing the correlation of metabolites among different samples (Fig. 7e). Notably, the hierarchical clustering analysis is consistent with the trend observed in PCA. In BL, high-concentration compounds include ethyl 3-methylbutyl acetate, ethyl hexanoate, 3-methylbutyl 3-methylbutanoate, ethyl octanoate, ethyl pentanoate, ethyl 2-hydroxypropanate, 4-hydroxybutan-2-one, acetic acid, and 2-hydroxypropanamide. For UBL, the compounds present in higher concentrations are ethyl butanoate, 3-methylbutan-1-ol, 1,1-dioxyethane, diethyl butanediate, ethyl 2-hydroxy-4-methylpentanoate, 3-methylbutyl 2-hydroxypropanate, 2-phenylethanol, 2,4-di-tert-butylphenol, (2E, 4E)-deca-2,4-dinal, ethyl propanate, 2-phenylethyl acetate, ethyl acetate, 1-O-ethyl 4-O-(3-methylbutyl) butanediate, ethyl (2*S*)-2-hydroxypropanate, and (E)-4-(2,6,6-triparate) methylcyclohexexen-1-yl) but-3-en-2-one. In UFW, high-concentration compounds include octanoic acid, decanoic acid, N-benzyl-N-(1-phenylethyl) nitroamide, ethyl dodecanoate, dec-9-enoic acid, hexanoic acid, 2,4,5-trimethyl-1,3-dioxolane, (2*R*,3*R*)-butane-2,3-diol, ethyl decanoate, and 2-hydroxypropanamide.

**Table 7:** Contents of characteristic volatile compounds in *Undaria* blended liquor and fermented wine.

Number	Compounds	RI	CAS No.	Formula	Content (mg/L)		
					BL	UBL	UFW
1	ethyl octanoate	1433	106–32-1	C_10_H_20_O_2_	844.09 ± 36.61^a^	363.47 ± 62.29^b^	251.46 ± 42.18^c^
2	ethyl acetate	887	141–78-6	C_4_H_8_O_2_	1.21 ± 0.14^b^	536.71 ± 34.05^a^	28.33 ± 0.93^b^
3	4-hydroxybutan-2-one	957	590–90-9	C_4_H_8_O_2_	308.54 ± 3.98^a^	–	–
4	diethyl butanedioate	1679	123–25-1	C_8_H_14_O_4_	154.5 ± 14.27^b^	299.55 ± 19.21^a^	–
5	decanoic acid	2274	334–48-5	C_10_H_20_O_2_	11.51 ± 1.61^b^	–	277.33 ± 27.63^a^
6	ethyl hexanoate	1232	123–66-0	C_8_H_16_O_2_	368.05 ± 7.82^a^	334.49 ± 42.27^a^	90.22 ± 2.69^b^
7	octanoic acid	2058	124–07-2	C_8_H_16_O_2_	10.85 ± 3.66^b^	18.68 ± 5.12^b^	261.58 ± 26.23^a^
8	ethyl (2*S*)-2-hydroxypropanoate	1347	687–47–8	C_5_H_10_O_3_	31.42 ± 0.5^b^	196.72 ± 41.59^a^	–
9	3-methylbutyl acetate	1121	123-92-2	C_10_H_20_O_2_	117.41 ± 13.71^a^	31.16 ± 0.86^b^	–
10	3-methylbutan-1-ol	1207	123–51-3	C_5_H_12_O	282.71 ± 34.86^b^	365.65 ± 2.07^a^	117.3 ± 14.09^c^
11	ethyl pentanoate	1134	539–82-2	C_7_H_14_O_2_	187.61 ± 26.72^a^	90.3 ± 13.89^b^	0.33 ± 0.03^c^
12	ethyl dodecanoate	1841	106–33-2	C_14_H_28_O_2_	–	–	168.28 ± 7.3^a^
13	acetic acid	1448	64–19-7	C_2_H_4_O_2_	111.32 ± 19.99^a^	–	10.82 ± 2.62^b^
14	ethyl propanoate	952	105–37-3	C_5_H_10_O_2_	–	109.52 ± 4.75^a^	–
15	3-methylbutyl 3-methylbutanoate	1292	659–70-1	C_10_H_20_O_2_	117.41 ± 13.71^a^	31.16 ± 0.86^b^	–
16	2-phenylethyl acetate	1811	103–45-7	C_10_H_12_O_2_	13.83 ± 1.04^b^	112.58 ± 3.89^a^	6.03 ± 0.09^c^
17	ethyl decanoate	1638	110–38-3	C_12_H_24_O_2_	62.66 ± 14.57^b^	19.57 ± 6.52^c^	164.53 ± 9.91^a^
18	ethyl 2-hydroxypropanoate	1345	97–64-3	C_5_H_10_O_3_	59.31 ± 1.56^a^	–	–
19	(E)-4-(2,6,6-trimethylcyclohexen-1-yl)but-3-en-2-one	1939	79–77-6	C_13_H_20_O	–	67.1 ± 7.19^a^	23.18 ± 2.33^b^
20	ethyl 2-hydroxy-4-methylpentanoate	1546	10,348–47-7	C_8_H_16_O_3_	43.82 ± 3.19^b^	97.1 ± 2.02^a^	–
21	1,1-diethoxyethane	890	105–57-7	C_6_H_14_O_2_	53.56 ± 7.3^b^	98.35 ± 4.82^a^	7.75 ± 2.83^c^
22	2,4-ditert-butylphenol	2319	96–76-4	C_14_H_22_O	15.88 ± 4.31^b^	55.99 ± 5.46^a^	11.66 ± 1.9^b^
23	3-methylbutyl 2-hydroxypropanoate	1577	19,329–89-6	C_8_H_16_O_3_	28.48 ± 1.06^b^	58.94 ± 10.47^a^	–
24	ethyl butanoate	1034	105–54-4	C_6_H_12_O_2_	44.26 ± 7.18^b^	62.4 ± 1.93^a^	4.05 ± 0.74^c^
25	N-benzyl-N-(1-phenylethyl)nitrous amide	1833	–	C_15_H_16_N_2_O	–	–	49.18 ± 3.84^a^
26	hexanoic acid	1844	142–62-1	C_6_H_12_O_2_	–	–	47.17 ± 6.52^a^
27	(2E,4E)-deca-2,4-dienal	1809	25,152–84-5	C_10_H_16_O	–	30.48 ± 1.78^a^	–
28	2-hydroxypropanamide	1333	2043–43-8	C_3_H_7_NO_2_	34.91 ± 4.86^a^	4.95 ± 1^b^	36.66 ± 0.55^a^
29	ethanol	932	64–17-5	C_2_H_6_O	50.97 ± 4.61^a^	39.5 ± 0.42^b^	11.09 ± 2.76^c^
30	dec-9-enoic acid	2344	14,436–32-9	C_10_H_18_O_2_	–	–	37.16 ± 4.02^a^
31	2,4,5-trimethyl-1,3-dioxolane	992	3299-32-9	C_6_H_12_O_2_	–	–	34.42 ± 0.92^a^
32	(2*R*,3*R*)-butane-2,3-diol	1554	24,347–58-8	C_4_H_10_O_2_	–	–	34.49 ± 6.64^a^
33	2-phenylethanol	1905	60–12-8	C_8_H_10_O	10.95 ± 1.53^b^	34.7 ± 0.84^a^	6.82 ± 0.91^c^
34	1-O-ethyl 4-O-(3-methylbutyl) butanedioate	1899	28,024–16-0	C_11_H_20_O_4_	–	24.9 ± 6.79^a^	0.91 ± 0.08^b^

Notes: Data are means ± standard deviation. Different letters (a-c) within each row are significantly different (*p* < 0.05). “– “, not detected.

### 3.4 Analysis of antioxidant activity

**Fig. 8:**
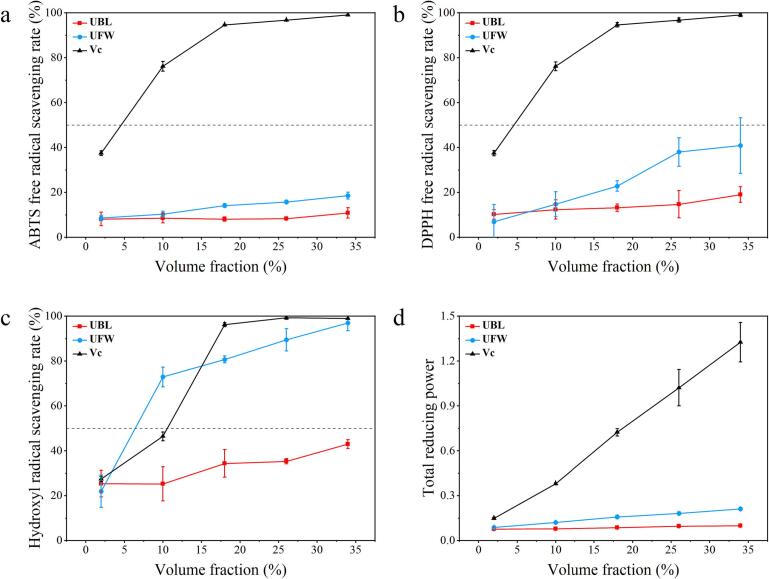
In vitro antioxidant test. The scavenging effect of the UBL, UFW and Vc on free radicals and the determination of total reducing power: (a) ABTS; (b) DPPH; (c) ·OH; (d) Total reducing power.

The in vitro antioxidant properties of UBL and UFW were assessed using four different assays: ABTS, DPPH, ·OH radical scavenging, and total reducing power. As depicted in Fig. 8, both UBL and UFW, along with Vc, demonstrated the ability to scavenge free radicals. The data showed that with the sample volume fraction increased, the scavenging activity of UFW on DPPH and ·OH radicals was significantly higher than that of UBL, demonstrating a strong dose-dependent relationship. The scavenging activity of UBL and UFW on ABTS radicals was low. The figure also shows that the reducing power of UBL and UFW increases with concentration, and UFW consistently outperforms UBL. At a 34 % volume fraction, the OD_700_ values for UBL and UFW were 0.098 and 0.21, respectively, indicating some antioxidant activity, although still lower than Vc, which had an OD_700_ of 1.33.

## 4 Discussion

Response surface methodology can be used to optimize the formulation of different foods, such as coffee-like maize beverage, wheat bread, Sandwich Cookie and greengage wines (Battaiotto et al., 2013; Collar et al., 1999; Tian et al., 2018; Youn & Chung, 2012). Process optimization is an effective strategy for improving target products. Research of Johnson et al. (2023) showed that compared with ordinary mulberry wine, Se-enriched mulberry wine optimized using RSM exhibited better physical and chemical properties, color, phytochemical content, antioxidant activity, and microbial quality. In this study, the ultrasonic parameters of UBL were optimized by RSM, and the highest PC was 0.66 g/L. The fermentation parameters of UFW were optimized to achieve the highest PC of 9.81 g/L. Under the same solid-liquid ratio, the PC of UFW was higher than that of UBL, which may be the result of the interaction between microorganisms and substrates during fermentation. Fermentation leads to the decomposition of complex substrates and/or biotransformation into compatible components, thereby regulating product characteristics or changing the number of certain bioactive compounds (Hussain et al., 2016).

Interestingly, TP were detected in UFW but not in UBL, which highlights the crucial role of fermentation in releasing or converting bioactive compounds from the substrate (De Villa et al., 2023). During the fermentation process, microorganisms facilitate the breakdown of complex molecules, enabling the release of phenolic compounds and their subsequent transformation into bioavailable or bioactive forms (De Villa et al., 2023). These bioactive components can significantly regulate the chemical composition, enrich the nutritional value, and enhance the health-promoting qualities of fermented products (Abbaspour, 2024; De Villa et al., 2023). Compounds such as (E)-4-(2,6,6-trimethylcyclohexen-1-yl)but-3-en-2-one (β-ionone), 4-(2,2,6-trimethyl-7-oxabicyclo[4.1.0]heptan-1-yl)but-3-en-2-one (β-Ionone epoxide) and 6-methylhept-5-en-2-one were detected in UBL and UFW (Supplementary Table S1). They may be derived from the biological components of *U. pinnatifida*. β-ionone and its derivatives can exhibit significant pharmacological activities, such as anti-inflammatory, anti-tumor, anti-fungal and antibacterial activities (Ansari & Emami, 2016). Its presence in UBL and UFW further proves that the ultrasonic process and the fermentation process can activate or enhance the bioactive components in the raw materials.

Non-targeted metabolomics aims to measure a broad spectrum of metabolites present in extracted samples without prior knowledge of their composition (Johnson et al., 2016). In this study, 34 characteristic volatile compounds were screened out by non-targeted metabolomics and multivariate statistical analysis (Table 7). Among them, the number of ester compounds is the largest. Coincidentally, esters are also the most abundant volatile components in pineapple wine, cherry wine and Chinese famous liquors, accounting for the largest proportion of the total concentration (Niu et al., 2011; Pino & Queris, 2010; Xiao et al., 2014). HCA highlighted the differences in the concentrations of volatile compounds across the three samples: BL, UBL, and UFW (Fig. 7e). These clusters aligned with the PCA results, confirming the similarity of their volatile compound profiles. For instance, BL contained high levels of ethyl 3-methylbutyl acetate, ethyl hexanoate, ethyl octanoate, and acetic acid, which are typically associated with fruity and floral aromas (González-Rodríguez et al., 2011; Xiao et al., 2014; Zheng et al., 2014). By contrast, UBL showed elevated levels of ester compounds such as ethyl butyrate, 3-methylbutanol, and diethyl succinate, which are main aroma-active components in aged Baijiu (Wang et al., 2021). UFW exhibited a distinct profile, with higher concentrations of fatty acids, including caprylic and capric acids, which imparted stronger earthy notes (Bossaert et al., 2019). Interestingly, shared esters between UBL and UFW suggest some overlap in their flavor characteristics despite differences in raw materials and production methods. Ethyl caprylate, ethyl acetate, and ethyl caproate, for example, are key flavor components in fermented beverages like whisky and rum (Nascimento et al., 2008), underscoring their role in shaping wine sensory profiles. The stability of wine flavours depends not only on the concentration of aroma compounds but also on wine composition (Styger et al., 2011). In addition to the influence of esters, alcohols, and acids, seaweed polysaccharides such as alginate may significantly enhance wine's physical stability. Alginate and its derivatives, known for their thickening and stabilising properties, are widely used in food as thickeners and emulsifiers (Himashree et al., 2022; Qin et al., 2018; Qin et al., 2020). Compared to agar, alginic acid and carrageenan likely provide greater stability, particularly in seaweed-fermented wines, enhancing texture and flavor persistence (Kraan, 2016). Understanding the complex interactions among volatile compounds, including esters, alcohols, and fatty acids, is essential for the sensory appeal of marine-fermented beverages. Future research should explore these interactions and their synergies with stabilisers like alginate to improve the quality and stability of these unique products.

The in vitro antioxidant capacity of UBL and UFW was assessed. Studies in the field of food science and nutrition have shown that antioxidants are beneficial for human health and help prevent chronic diseases (Willcox et al., 2004). In vitro results demonstrated that UBL and UFW possess antioxidant capacity with a dose-dependent effect. UFW exhibited stronger scavenging effects on ABTS, DPPH, hydroxyl radicals, and total reducing power compared to UBL. It is well known that fermentation can improve the bioavailability of bioactive compounds and stimulate the production of new bioactive metabolites (Septembre-Malaterre et al., 2018). The results of Ooi et al. (2020) showed that the application of screened yeast starter cultures in cocoa fermentation had a higher total polyphenol and flavonoid content in cocoa beans compared with the control at a specific fermentation period. Therefore, fermentation with *Undaria* as a substrate may enhance the nutritional value and functional properties, as evidenced by the significant antioxidant activity observed in vitro.

## 5 Conclusion

In this study, RSM was used to optimize the ultrasonic conditions of UBL and the fermentation conditions of UFW, and the model fitting results were satisfactory. After UBL process optimization, PC was 0.66 g/L and AC was 39.2 % vol. After UFW process optimization, PC was 9.81 g/L and AC was 8.3 % vol. The volatile compounds in BL, UBL and UFW were analyzed and compared, showing significant differences in the compounds and their concentrations (*p* < 0.05). Multivariate statistical analysis identified 34 characteristic volatile compounds in BL, UBL, and UFW, with 14, 15, and 23 volatile compounds quantified, respectively. Among these, esters were the most abundant, accounting for half. The in vitro antioxidant results indicated that bioactive components, such as polysaccharides, contributed to the antioxidant capacity of UBL and UFW, highlighting the potential of *Undaria pinnatifida* as a novel product with possible applications in specific liquor and wine markets.

## CRediT authorship contribution statement

**Fangru Nan:** Writing – review & editing, Writing – original draft, Investigation, Funding acquisition, Formal analysis. **Xinyi Li:** Writing – review & editing, Writing – original draft, Formal analysis, Data curation, Conceptualization. **Jia Feng:** Methodology, Investigation. **Junping Lv:** Project administration. **Qi Liu:** Project administration. **Xudong Liu:** Resources. **Yang Liu:** Supervision. **Ruikai Zhang:** Software. **Baoqing Bai:** Supervision. **Shulian Xie:** Writing – review & editing, Writing – original draft, Visualization, Investigation.

## Funding

This work was supported by the Open Project Program of Xinghuacun College of Shanxi University (Shanxi Institute of Brewing Technology and Industry) (No. XCSXU-KF-202221), the National Natural Science Foundation of China (No. 31800172 to F.R. Nan, No. 32170204 to S.L. Xie, No. 32270220 and U22A20445 to J. Feng) and the Excellent Achievement Cultivation Project of Higher education in Shanxi (No. 2020KJ029), the Water Conservancy Science and Technology Research and Promotion Projects in Shanxi province (2025GM38).

## Declaration of competing interest

The authors declare that they have no known competing financial interests or personal relationships that could have appeared to influence the work reported in this paper.

## Data Availability

Data will be made available on request.

## Appendix A Supplementary data

Supplementary material Image 1
