# Supplementary material for: Production, characterization and antioxidant analysis on the Undaria-based alcoholic beverages using response surface method and HS-SPME-GC × GC-TOF-MS
Source: Food Chem X. 2025 Apr 1;27:102428. doi: 10.1016/j.fochx.2025.102428 (PMC12002601; doi:10.1016/j.fochx.2025.102428)
Supplement: Supplementary file 1 — Supplementary material [file mmc1.docx]

**Supplementary Table S1**

Contents of volatile compounds in *Undaria* blended liquor and fermented wine

| Number | Compounds | RI | CAS No. | Formula | Content (mg/L) | | | |
| --- | --- | --- | --- | --- | --- | --- | --- | --- |
|  |  |  |  |  | BL | UBL | UFW |  |
| Esters |  |  |  |  |  |  |  |  |
| 1 | (3-hydroxy-2,2,4-trimethylpentyl) 2-methylpropanoate | 1869 | 77-68-9 | C_12_H_24_O_3_ | - | - | 20.61±1.81^a^ |  |
| 2 | [(2E,4E)-octa-2,4-dienyl] acetate | 1481 | - | C_10_H_16_O_2_ | - | - | 0.09±0.03^a^ |  |
| 3 | [2,2,4-trimethyl-3-(2-methylpropanoyloxy)pentyl] 2-methylpropanoate | 1855 | 6846-50-0 | C_16_H_30_O_4_ | - | - | 26.66±0.25^a^ |  |
| 4 | 1-acetyloxyethyl acetate | 1512 | 542-10-9 | C_6_H_10_O_4_ | 1.83±0.56^a^ | - | - |  |
| 5 | 1-hydroxypropan-2-yl acetate | 1617 | 6214-01-3 | C_5_H_10_O_3_ | - | 6.26±0.05^a^ | - |  |
| 6 | 1-O-ethyl 4-O-(3-methylbutyl) butanedioate | 1899 | 28024-16-0 | C_11_H_20_O_4_ | - | 24.9±6.79^a^ | 0.91±0.08^b^ |  |
| 7 | 2-ethoxyethyl octanoate | 1998 | - | C_12_H_24_O_3_ | - | - | 1.44±0.11^a^ |  |
| 8 | 2-methylpropyl acetate | 1010 | 110-19-0 | C_6_H_12_O_2_ | 5±0.81^b^ | 9.54±1.69^a^ | 0.44±0.07^c^ |  |
| 9 | 2-methylpropyl decanoate | 1748 | 30673-38-2 | C_14_H_28_O_2_ | - | - | 0.78±0.13^a^ |  |
| 10 | 2-methylpropyl hexanoate | 1350 | 105-79-3 | C_10_H_20_O_2_ | - | - | 0.18±0.02^a^ |  |
| 11 | 2-methylpropyl octanoate | 1546 | 5461-06-3 | C_12_H_24_O_2_ | - | - | 0.38±0.02^a^ |  |
| 12 | 2-phenylethyl 2-methylpropanoate | 1896 | 103-48-0 | C_12_H_16_O_2_ | - | - | 0.34±0.21^a^ |  |
| 13 | 2-phenylethyl acetate | 1811 | 103-45-7 | C_10_H_12_O_2_ | 13.83±1.04^b^ | 112.58±3.89^a^ | 6.03±0.09^c^ |  |
| 14 | 2-propylpentyl formate | 1711 | - | C_9_H_18_O_2_ | - | 12.16±0.48^a^ | - |  |
| 15 | 3-methylbutyl 2-hydroxypropanoate | 1577 | 19329-89-6 | C_8_H_16_O_3_ | 28.48±1.06^b^ | 58.94±10.47^a^ | - |  |
| 16 | 3-methylbutyl 2-methylbutanoate | 1275 | 27625-35-0 | C_10_H_20_O_2_ | 19.87±2.67^a^ | 1.52±0.98^b^ | - |  |
| 17 | 3-methylbutyl 2-methylpropanoate | 1192 | 2050-01-3 | C_9_H_18_O_2_ | - | 0.28±0.16^a^ | - |  |
| 18 | 3-methylbutyl 3-methylbutanoate | 1292 | 659-70-1 | C_10_H_20_O_2_ | 117.41±13.71^a^ | 31.16±0.86^b^ | - |  |
| 19 | 3-methylbutyl acetate | 1121 | 123-92-2 | C_7_H_14_O_2_ | 244.37±13.39^a^ | 171.84±12.8^b^ | 18.13±2.53^c^ |  |
| 20 | 3-methylbutyl butanoate | 1257 | 106-27-4 | C_9_H_18_O_2_ | 13.93±1.68^a^ | 3.05±1.45^b^ | - |  |
| 21 | 3-methylbutyl decanoate | 1861 | 2306-91-4 | C_15_H_30_O_2_ | - | - | 3.74±0.85^a^ |  |
| 22 | 3-methylbutyl hexanoate | 1450 | 2198-61-0 | C_11_H_22_O_2_ | - | - | 0.87±0^a^ |  |
| 23 | 3-methylbutyl octanoate | 1656 | 2035-99-6 | C_13_H_26_O_2_ | - | - | 6.79±0.59^a^ |  |
| 24 | 3-methylbutyl pentanoate | 1348 | 2050-09-1 | C_10_H_20_O_2_ | 0.52±0.24^a^ | - | - |  |
| 25 | 3-methylbutyl propanoate | 1183 | 105-68-0 | C_8_H_16_O_2_ | 1.15±0.25^a^ | - | - |  |
| 26 | 3-oxobutan-2-yl acetate | 1376 | 4906-24-5 | C_6_H_10_O_3_ | 3.34±0.97^a^ | - | - |  |
| 27 | 5-[(Z)-oct-2-enyl]oxolan-2-one | 2394 | 18679-18-0 | C_12_H_20_O_2_ | - | - | 0.59±0.02^a^ |  |
| 28 | bis(2-methylpropyl) benzene-1,2-dicarboxylate | 2534 | 84-69-5 | C_16_H_22_O_4_ | - | 0.42±0.02^a^ | - |  |
| 29 | butan-2-yl acetate | 986 | 105-46-4 | C_6_H_12_O_2_ | 0.37±0.27^a^ | 0.29±0^ab^ | - |  |
| 30 | butyl acetate | 1073 | 123-86-4 | C_6_H_12_O_2_ | 0.54±0.03^b^ | 18.49±4.68^a^ | - |  |
| 31 | butyl butanoate | 1218 | 109-21-7 | C_8_H_16_O_2_ | 0.48±0.11^b^ | 3.11±0.14^a^ | - |  |
| 32 | dibutyl benzene-1,2-dicarboxylate | 2679 | 84-74-2 | C_16_H_22_O_4_ | 6.48±0.44^a^ | - | 1.31±0.28^b^ |  |
| 33 | diethyl butanedioate | 1679 | 123-25-1 | C_8_H_14_O_4_ | 154.5±14.27^b^ | 299.55±19.21^a^ | - |  |
| 34 | diethyl nonanedioate | 2233 | 624-17-9 | C_13_H_24_O_4_ | - | 4.25±1.03^a^ | - |  |
| 35 | ethyl (2S)-2-hydroxypropanoate | 1347 | 687-47-8 | C_5_H_10_O_3_ | 31.42±0.5^b^ | 196.72±41.59^a^ | - |  |
| 36 | ethyl (E)-dec-2-enoate | 1750 | 7367-88-6 | C_12_H_22_O_2_ | - | - | 0.33±0.05^a^ |  |
| 37 | ethyl (E)-hept-3-enoate | 1386 | 54340-71-5 | C_9_H_16_O_2_ | - | 0.52±0.29^a^ | - |  |
| 38 | ethyl (E)-hept-4-enoate | 1380 | 54340-70-4 | C_9_H_16_O_2_ | - | 1.32±0^a^ | 0.36±0.06^b^ |  |
| 39 | ethyl (E)-hept-5-enoate | 1375 | 54340-69-1 | C_9_H_16_O_2_ | - | - | 0.29±0.16^a^ |  |
| 40 | ethyl (E)-hex-2-enoate | 1328 | 27829-72-7 | C_8_H_14_O_2_ | - | - | 0.39±0.17^a^ |  |
| 41 | ethyl (E)-hex-3-enoate | 1290 | 2396-83-0 | C_8_H_14_O_2_ | - | 7.21±1.09^a^ | - |  |
| 42 | ethyl (E)-oct-2-enoate | 1540 | 7367-82-0 | C_10_H_18_O_2_ | - | - | 0.68±0.02^a^ |  |
| 43 | ethyl 2-[(E)-prop-1-enyl]cyclopropane-1-carboxylate | 1631 | - | C_9_H_14_O_2_ | - | - | 0.46±0.02^a^ |  |
| 44 | ethyl 2-chloropropanoate | 1248 | 535-13-7 | C_5_H_9_ClO_2_ | 0.21±0.05^a^ | - | - |  |
| 45 | ethyl 2-hydroxy-3-methylbutanoate | 1420 | 2441-06-7 | C_7_H_14_O_3_ | 7.85±1.54^a^ | 2.06±1.07^b^ | - |  |
| 46 | ethyl 2-hydroxy-4-methylpentanoate | 1546 | 10348-47-7 | C_8_H_16_O_3_ | 43.82±3.19^b^ | 97.1±2.02^a^ | - |  |
| 47 | ethyl 2-hydroxybutanoate | 1399 | 52089-54-0 | C_6_H_12_O_3_ | 0.77±0.28^a^ | - | - |  |
| 48 | ethyl 2-hydroxypropanoate | 1345 | 97-64-3 | C_5_H_10_O_3_ | 59.31±1.56^a^ | - | - |  |
| 49 | ethyl 2-methylbutanoate | 1050 | 7452-79-1 | C_7_H_14_O_2_ | 3.51±0.6^b^ | 6.86±1.52^a^ | - |  |
| 50 | ethyl 2-methylpropanoate | 960 | 97-62-1 | C_6_H_12_O_2_ | 7.51±2.18^b^ | 26.57±7.33^a^ | - |  |
| 51 | ethyl 2-phenylacetate | 1783 | 101-97-3 | C_10_H_12_O_2_ | 5.89±0.37^b^ | 23.7±5.08^a^ | - |  |
| 52 | ethyl 3-hydroxytridecanoate | 2431 | 107141-15-1 | C_15_H_30_O_3_ | - | - | 4.32±0.28^a^ |  |
| 53 | ethyl 3-methylbutanoate | 1066 | 108-64-5 | C_7_H_14_O_2_ | 7.98±1.69^b^ | 16.05±4.71^a^ | 0.1±0.02^c^ |  |
| 54 | ethyl 3-phenylpropanoate | 1891 | 2021-28-5 | C_11_H_14_O_2_ | 4.95±0.55^a^ | - | - |  |
| 55 | ethyl 4-methylpentanoate | 1188 | 25415-67-2 | C_8_H_16_O_2_ | - | 1.35±0.49^a^ | - |  |
| 56 | ethyl 5-methylhexanoate | 1288 | 10236-10-9 | C_9_H_18_O_2_ | 0.21±0.04^a^ | - | - |  |
| 57 | ethyl acetate | 887 | 141-78-6 | C_4_H_8_O_2_ | 1.21±0.14^b^ | 536.71±34.05^a^ | 28.33±0.93^b^ |  |
| 58 | ethyl butanoate | 1034 | 105-54-4 | C_6_H_12_O_2_ | 44.26±7.18^b^ | 62.4±1.93^a^ | 4.05±0.74^c^ |  |
| 59 | ethyl dec-9-enoate | 1692 | 67233-91-4 | C_12_H_22_O_2_ | - | - | 22.27±0.94^a^ |  |
| 60 | ethyl decanoate | 1638 | 110-38-3 | C_12_H_24_O_2_ | 62.66±14.57^b^ | 19.57±6.52^c^ | 164.53±9.91^a^ |  |
| 61 | ethyl dodecanoate | 1841 | 106-33-2 | C_14_H_28_O_2_ | - | - | 168.28±7.3^a^ |  |
| 62 | ethyl hept-6-enoate | 1607 | 25118-23-4 | C_9_H_16_O_2_ | 0.23±0.04^a^ | - | - |  |
| 63 | ethyl heptanoate | 1329 | 106-30-9 | C_9_H_18_O_2_ | 15.46±2.08^a^ | 17.31±5.27^a^ | 1.34±0.03^b^ |  |
| 64 | ethyl hexadecanoate | 2249 | 628-97-7 | C_18_H_36_O_2_ | 2.65±1.35^b^ | 1.5±0.44^b^ | 6.59±1.14^a^ |  |
| 65 | ethyl hexanoate | 1232 | 123-66-0 | C_8_H_16_O_2_ | 368.05±7.82^a^ | 334.49±42.27^a^ | 90.22±2.69^b^ |  |
| 66 | ethyl nonanoate | 1530 | 123-29-5 | C_11_H_22_O_2_ | 6.17±0.99^a^ | 4.15±2.52^a^ | 0.76±0.03^b^ |  |
| 67 | ethyl nonyl carbonate | 1633 | - | C_12_H_24_O_3_ | 8.12±1.69^a^ | - | - |  |
| 68 | ethyl octanoate | 1433 | 106-32-1 | C_10_H_20_O_2_ | 844.09±36.61^a^ | 363.47±62.29^b^ | 251.46±42.18^c^ |  |
| 69 | ethyl pentanoate | 1134 | 539-82-2 | C_7_H_14_O_2_ | 187.61±26.72^a^ | 90.3±13.89^b^ | 0.33±0.03^c^ |  |
| 70 | ethyl propanoate | 952 | 105-37-3 | C_5_H_10_O_2_ | - | 109.52±4.75^a^ | - |  |
| 71 | ethyl tetradecanoate | 2048 | 124-06-1 | C_16_H_32_O_2_ | - | - | 11.94±1.86^a^ |  |
| 72 | ethyl undecanoate | 1737 | 627-90-7 | C_13_H_26_O_2_ | - | - | 1.35±0.17^a^ |  |
| 73 | heptyl formate | 1355 | 112-23-2 | C_8_H_16_O_2_ | - | - | 13.54±2.01^a^ |  |
| 74 | methyl 2-hydroxy-2-methylbutanoate | 1275 | 32793-34-3 | C_6_H_12_O_3_ | - | 0.07±0.01^a^ | - |  |
| 75 | pentyl acetate | 1174 | 628-63-7 | C_7_H_14_O_2_ | - | 0.18±0.06^a^ | - |  |
| 76 | propyl acetate | 971 | 109-60-4 | C_5_H_10_O_2_ | 10.21±1.69^a^ | 7.71±1.82^a^ | - |  |
| 77 | propyl hexanoate | 1314 | 626-77-7 | C_9_H_18_O_2_ | 0.26±0.07^a^ | - | - |  |
| **Alcohols** |  |  |  |  |  |  |  |  |
| 78 | (2R,3R)-butane-2,3-diol | 1554 | 24347-58-8 | C_4_H_10_O_2_ | - | - | 34.49±6.64^a^ |  |
| 79 | (2S)-heptan-2-ol | 1322 | 6033-23-4 | C_7_H_16_O | 0.22±0.1^a^ | - | - |  |
| 80 | (3E,6E)-nona-3,6-dien-1-ol | 1745 | [56805-23-3](https://pubchem.ncbi.nlm.nih.gov/compound/44630408) | C_9_H_16_O | - | - | 1.19±0.3^a^ |  |
| 81 | (3R)-3,7-dimethyloct-6-en-1-ol | 1766 | 1117-61-9 | C_10_H_20_O | - | - | 1.55±0.2^a^ |  |
| 82 | (6E)-3,7,11-trimethyldodeca-1,6,10-trien-3-ol | 2040 | 7212-44-4 | C_15_H_26_O | - | - | 4.52±0.1^a^ |  |
| 83 | (E)-non-6-en-1-ol | 1730 | 31502-19-9 | C_9_H_18_O | - | - | 1.09±0.45^a^ |  |
| 84 | (Z)-dec-3-en-1-ol | 1787 | 10340-22-4 | C_10_H_20_O | - | 10.43±1.31^a^ | - |  |
| 85 | (Z)-dec-5-en-1-ol | 1808 | 51652-47-2 | C_10_H_20_O | - | - | 1.15±0.69^a^ |  |
| 86 | (Z)-oct-5-en-1-ol | 1613 | 64275-73-6 | C_8_H_16_O | - | - | 3.04±0.2^a^ |  |
| 87 | 1,3,3-trimethyl-2-oxabicyclo[2.2.2]octan-6-ol | 1843 | 18679-48-6 | C_10_H_18_O_2_ | - | - | 0.67±0.04^a^ |  |
| 88 | 1-[2-Methyl-2-(4-methyl-3-pentenyl)cyclopropyl]ethanol | 2371 | - | C_12_H_22_O | 1.57±0.03^a^ | - | - |  |
| 89 | 2-(2,6,6-trimethylcyclohexen-1-yl)ethanol | 1933 | 472-65-1 | C_11_H_20_O | - | - | 1.31±0.12^a^ |  |
| 90 | 2,5-dimethylhexane-2,5-diol | 1819 | 110-03-2 | C_8_H_18_O_2_ | 4.79±1.81^a^ | 2.36±0.5^b^ | 0.94±0.14^b^ |  |
| 91 | 2-[2-[2-[2-(2-hydroxyethoxy)ethoxy]ethoxy]ethoxy]ethanol | 2115 | 4792-15-8 | C_10_H_22_O_6_ | - | 2.06±0.27^a^ | - |  |
| 92 | 2-methylpropan-1-ol | 1090 | 78-83-1 | C_4_H_10_O | 46.62±7.4^a^ | 55.52±1.84^a^ | 21.91±2.22^b^ |  |
| 93 | 2-methylpropan-2-ol | 898 | 75-65-0 | C_4_H_10_O | 2.7±0.12^b^ | 8.54±1.35^a^ | 1.35±0.01^b^ |  |
| 94 | 2-phenylethanol | 1905 | 60-12-8 | C_8_H_10_O | 10.95±1.53^b^ | 34.7±0.84^a^ | 6.82±0.91^c^ |  |
| 95 | 3,7-dimethylocta-1,6-dien-3-ol | 1545 | 78-70-6 | C_10_H_18_O | - | - | 0.51±0.01^a^ |  |
| 96 | 3-methylbutan-1-ol | 1207 | 123-51-3 | C_5_H_12_O | 282.71±34.86^b^ | 365.65±2.07^a^ | 117.3±14.09^c^ |  |
| 97 | 3-methylsulfanylpropan-1-ol | 1717 | 505-10-2 | C_4_H_10_OS | - | - | 0.8±0.01^a^ |  |
| 98 | 7-methyl-3-methylideneoct-6-en-1-ol | 1798 | 13066-51-8 | C_10_H_18_O | - | - | 0.55±0.14^a^ |  |
| 99 | butan-1-ol | 1140 | 71-36-3 | C_4_H_10_O | - | 6.92±1.01^a^ | - |  |
| 100 | butan-2-ol | 1023 | 78-92-2 | C_4_H_10_O | 2.17±0.53^a^ | ^-^ | - |  |
| 101 | dec-9-yn-1-ol | 1964 | 17643-36-6 | C_10_H_18_O | - | - | 12.08±0.52^a^ |  |
| 102 | deca-2,4-dien-1-ol | 1957 | [16195-71-4](https://pubchem.ncbi.nlm.nih.gov/compound/87632) | C_10_H_18_O | - | 3.87±0.16^a^ | - |  |
| 103 | decan-1-ol | 1758 | 112-30-1 | C_10_H_22_O | - | - | 0.55±0.01^a^ |  |
| 104 | ethanol | 932 | 64-17-5 | C_2_H_6_O | 50.97±4.61^a^ | 39.5±0.42^b^ | 11.09±2.76^c^ |  |
| 105 | hexan-1-ol | 1353 | 111-27-3 | C_6_H_14_O | - | - | 2.13±0.11^a^ |  |
| 106 | nonan-1-ol | 1658 | 143-08-8 | C_9_H_20_O | 0.84±0.15^b^ | 20.43±0.98^a^ | 1.45±0.2^b^ |  |
| 107 | nonan-2-ol | 1519 | 628-99-9 | C_9_H_20_O | - | - | 0.26±0.02^a^ |  |
| 108 | oct-1-en-3-ol | 1448 | 3391-86-4 | C_8_H_16_O | - | - | 4.7±0.46^a^ |  |
| 109 | octan-1-ol | 1555 | 111-87-5 | C_8_H_18_O | - | - | 6.32±0.7^a^ |  |
| 110 | octan-3-ol | 1391 | 589-98-0 | C_8_H_18_O | - | 1.71±0.46^a^ | - |  |
| 111 | oxiran-2-ylmethanol | 988 | 556-52-5 | C_3_H_6_O_2_ | 0.93±0.09^a^ | 0.05±0.02^b^ | - |  |
| 112 | propan-1-ol | 1034 | 71-23-8 | C_3_H_8_O | 28.55±2.31^a^ | 34.42±8.53^a^ | 2.41±0.36^b^ |  |
| 113 | undec-10-en-1-ol | 1662 | 112-43-6 | C_11_H_22_O | - | - | 2.71±0.36^a^ |  |
| **Acids** |  |  |  |  |  |  |  |  |
| 114 | (8E,11E,14E)-icosa-8,11,14-trienoic acid | 2213 | 7324-41-6 | C_20_H_34_O_2_ | - | - | 0.7±0.22^a^ |  |
| 115 | 3,4-bis(methoxycarbonyl)benzoic acid | 855 | 54699-35-3 | C_11_H_10_O_6_ | 0.43±0.08^a^ | - | - |  |
| 116 | 8-chlorooctanoic acid | 2033 | 1795-62-6 | C_8_H_15_ClO_2_ | - | - | 5.73±1.23^a^ |  |
| 117 | acetic acid | 1447 | 64-19-7 | C_2_H_4_O_2_ | 111.32±19.99^a^ | - | 10.82±2.62^b^ |  |
| 118 | dec-9-enoic acid | 2344 | 14436-32-9 | C_10_H_18_O_2_ | - | - | 37.16±4.02^a^ |  |
| 119 | decanoic acid | 2274 | 334-48-5 | C_10_H_20_O_2_ | 11.51±1.61^b^ | - | 277.33±27.63^a^ |  |
| 120 | dodecanoic acid | 2494 | 143-07-7 | C_12_H_24_O_2_ | - | - | 6.11±0.44^a^ |  |
| 121 | heptanoic acid | 1948 | 111-14-8 | C_7_H_14_O_2_ | - | - | 2.04±0.51^a^ |  |
| 122 | hexanoic acid | 1844 | 142-62-1 | C_6_H_12_O_2_ | - | - | 47.17±6.52^a^ |  |
| 123 | nonanoic acid | 2168 | 112-05-0 | C_9_H_18_O_2_ | 4.45±0.96^b^ | - | 6.87±1^a^ |  |
| 124 | octanoic acid | 2058 | 124-07-2 | C_8_H_16_O_2_ | 10.85±3.66^b^ | 18.68±5.12^b^ | 261.58±26.23^a^ |  |
| **Aldehydes** |  |  |  |  |  |  |  |  |
| 125 | (2E,4E)-deca-2,4-dienal | 1809 | 25152-84-5 | C_10_H_16_O | - | 30.48±1.78^a^ | - |  |
| 126 | (2E,4E)-hepta-2,4-dienal | 1493 | 4313-03-5 | C_7_H_10_O | - | - | 0.3±0.07^a^ |  |
| 127 | (7Z,10Z,13Z)-hexadeca-7,10,13-trienal | 1592 | - | C_16_H_26_O | - | 2.99±0.92^a^ | - |  |
| 128 | (E)-dec-2-enal | 1642 | 3913-81-3 | C_10_H_18_O | - | - | 1.47±0.12^a^ |  |
| 129 | (E)-non-2-enal | 1532 | 18829-56-6 | C_9_H_16_O | - | 5.94±1.45^a^ | - |  |
| 130 | (E)-oct-2-enal | 1427 | 2548-87-0 | C_8_H_14_O | - | 4.93±1.18^a^ | 0.65±0.12^b^ |  |
| 131 | 2,6,6-trimethylcyclohexa-1,3-diene-1-carbaldehyde | 1614 | 116-26-7 | C_10_H_14_O | - | - | 0.72±0.03^a^ |  |
| 132 | 2,6,6-trimethylcyclohexene-1-carbaldehyde | 1609 | 432-25-7 | C_10_H_16_O | - | 3.93±0.67^a^ | 3.17±0.22^a^ |  |
| 133 | 2-butyl-2-ethyl-5-methyl-hexa-3,4-dienal | 1463 | 23739-80-2 | C_13_H_22_O | - | - | 10.8±1.76^a^ |  |
| 134 | 3-methylbutanal | 916 | 590-86-3 | C_5_H_10_O | 9.31±1.16^b^ | 16.8±2.55^a^ | - |  |
| 135 | acetaldehyde | 700 | 75-07-0 | C_2_H_4_O | 1.19±0.57^a^ | 0.54±0.33^ab^ | - |  |
| 136 | decanal | 1496 | 112-31-2 | C_10_H_20_O | - | 2.04±0.63^a^ | 0.93±0.54^b^ |  |
| 137 | dodecanal | 1709 | 112-54-9 | C_12_H_24_O | - | - | 0.59±0.23^a^ |  |
| 138 | hexanal | 1081 | 66-25-1 | C_6_H_12_O | 2.54±0.15^b^ | 20.1±5.08^a^ | 1.14±0.23^b^ |  |
| 139 | nonanal | 1389 | 124-19-6 | C_9_H_18_O | 7.71±0.62^b^ | 10.43±1.48^a^ | 2.19±0.58^c^ |  |
| 140 | undecanal | 1602 | 112-44-7 | C_11_H_22_O | - | - | 1.54±0.01^a^ |  |
| **Ketones** |  |  |  |  |  |  |  |  |
| 141 | (2Z)-2-(2-methylpropylidene)cycloheptan-1-one | 1606 | - | C_11_H_18_O | - | - | 0.49±0.04^a^ |  |
| 142 | (3E,5E)-octa-3,5-dien-2-one | 1568 | 38284-27-4 | C_8_H_12_O | - | 11.27±0.23^a^ | - |  |
| 143 | (5E)-6,10-dimethylundeca-5,9-dien-2-one | 1857 | 3796-70-1 | C_13_H_22_O | - | - | 1.25±0.18^a^ |  |
| 144 | (7aR)-4,4,7a-trimethyl-6,7-dihydro-5H-1-benzofuran-2-one | 2329 | 17092-92-1 | C_11_H_16_O_2_ | - | 1.15±0.24^b^ | 4.39±0.13^a^ |  |
| 145 | (E)-1-(2,6,6-trimethylcyclohexa-1,3-dien-1-yl)but-2-en-1-one | 1821 | 23696-85-7 | C_13_H_18_O | 1.12±0.3^b^ | 4.48±1.14^a^ | - |  |
| 146 | (E)-4-(2,6,6-trimethylcyclohexen-1-yl)but-3-en-2-one | 1939 | 79-77-6 | C_13_H_20_O | - | 67.1±7.19^a^ | 23.18±2.33^b^ |  |
| 147 | (Z)-1-(1,2,2-trimethylcyclopentyl)pent-2-ene-1,4-dione | 2142 | - | C_13_H_20_O_2_ | - | - | 0.37±0.2^a^ |  |
| 148 | (Z)-oct-6-en-2-one | 1316 | - | C_8_H_14_O | - | - | 1.91±0.71^a^ |  |
| 149 | 1-(1,1-dimethyl-2,3-dihydroinden-4-yl)ethanone | 2536 | - | C_13_H_16_O | - | - | 0.95±0.13^a^ |  |
| 150 | 1-(4,6,8-trimethylazulen-1-yl)ethanone | 1837 | - | C_15_H_16_O | - | 2.42±0.64^b^ | 5.13±0.95^a^ |  |
| 151 | 1-cyclohexylbutan-1-one | 1982 | 1462-27-7 | C_10_H_18_O | - | - | 7.85±1.17^a^ |  |
| 152 | 2,2,6-trimethylcyclohexan-1-one | 1315 | 2408-37-9 | C_9_H_16_O | - | 1.01±0.1^a^ | 0.19±0.01^b^ |  |
| 153 | 2-methylheptan-3-one | 1177 | 13019-20-0 | C_8_H_16_O | - | 1.79±0.39^a^ | - |  |
| 154 | 4-(2,2,6-trimethyl-7-oxabicyclo[4.1.0]heptan-1-yl)but-3-en-2-one | 1960 | 23267-57-4 | C_13_H_20_O_2_ | - | 7.51±1.18^a^ | 2.12±0.61^b^ |  |
| 155 | 4-hydroxybutan-2-one | 957 | 590-90-9 | C_4_H_8_O_2_ | 308.54±3.98^a^ | - | - |  |
| 156 | 5-pentyloxolan-2-one | 2022 | 104-61-0 | C_9_H_16_O_2_ | 1.21±0.28^b^ | - | 6.4±1.34^a^ |  |
| 157 | 6-heptyloxan-2-one | 2424 | 713-95-1 | C_12_H_22_O_2_ | - | - | 0.91±0.54^a^ |  |
| 158 | 6-methylhept-5-en-2-one | 1337 | 110-93-0 | C_8_H_14_O | - | 1.14±0.31^a^ | 0.58±0.02^b^ |  |
| 159 | 6-methylheptan-2-one | 1235 | 928-68-7 | C_8_H_16_O | - | 0.29±0.17^a^ | - |  |
| 160 | butane-2,3-dione | 977 | 431-03-8 | C_4_H_6_O_2_ | - | - | 0.49±0.14^a^ |  |
| 161 | heptan-2-one | 1180 | 110-43-0 | C_7_H_14_O | - | 5.24±0.83^a^ | 0.42±0.04^b^ |  |
| 162 | nonan-2-one | 1388 | 821-55-6 | C_9_H_18_O | - | - | 0.34±0.1^a^ |  |
| 163 | octan-2-one | 1285 | 111-13-7 | C_8_H_16_O | - | 3.98±0.28^a^ | - |  |
| 164 | pentan-2-one | 980 | 107-87-9 | C_5_H_10_O | - | - | 0.44±0.02^a^ |  |
| 165 | propan-2-one | 817 | 67-64-1 | C_3_H_6_O | 29.01±1.02^a^ | 18.47±3.67^b^ | 4.27±1.37^c^ |  |
| **Hydrocarbons** |  |  |  |  |  |  |  |  |
| 166 | tetradec-1-ene | 1441 | 1120-36-1 | C_14_H_28_ | 0.35±0.04^a^ | - | - |  |
| 167 | (E)-3,5,5-trimethylhex-2-ene | 1433 | 26456-76-8 | C_9_H_18_ | - | - | 1.93±0.61^a^ |  |
| 168 | 1,1,5-trimethyl-2H-naphthalene | 1754 | - | C_13_H_16_ | - | - | 0.2±0.01^a^ |  |
| 169 | 1,2-xylene | 1185 | 95-47-6 | C_8_H_10_ | - | - | 0.52±0.03^a^ |  |
| 170 | 10-methoxydec-1-ene | 1723 | 72928-41-7 | C_11_H_22_O | - | - | 6.58±0.62^a^ |  |
| 171 | 1-methyl-3-propan-2-ylbenzene | 1267 | 535-77-3 | C_10_H_14_ | - | - | 2.41±0.44^a^ |  |
| 172 | 4,7-dimethylundecane | 1258 | 17301-32-5 | C_13_H_28_ | 0.88±0.11^a^ | 0.33±0.02^b^ | - |  |
| 173 | 5-ethylnon-1-ene | 1777 | 19780-74-6 | C_11_H_22_ | - | - | 3.29±0.25^a^ |  |
| 174 | 6-ethyl-2-methyldecane | 1388 | 62108-21-8 | C_13_H_28_ | 0.33±0.19^b^ | 0.97±0.11^a^ | - |  |
| 175 | dodec-3-yne | 1397 | 6790-27-8 | C_12_H_22_ | - | - | 4.24±1.61^a^ |  |
| 176 | hexadecane | 1598 | 544-76-3 | C_16_H_34_ | 0.49±0.09^b^ | - | 1.66±0.02^a^ |  |
| 177 | undec-2-yne | 1360 | 60212-29-5 | C_11_H_20_ | - | 9.04±1.72^a^ | - |  |
| 178 | undecane | 1098 | 1120-21-4 | C_11_H_24_ | - | 0.24±0.01^a^ | - |  |
| 179 | styrene | 1259 | 100-42-5 | C_8_H_8_ | 0.29±0.03^a^ | 0.4±0.1^a^ | - |  |
| 180 | (3E,5Z)-octa-1,3,5-triene | 1108 | 33580-05-1 | C_8_H_12_ | - | 0.55±0.08^a^ | - |  |
| 181 | (4Z,6Z,9Z)-nonadeca-4,6,9-triene | 1881 | 874302-34-8 | C_19_H_34_ | - | - | 0.77±0.52^a^ |  |
| 182 | 1,2,4,5-tetramethylbenzene | 1431 | 95-93-2 | C_10_H_14_ | - | - | 1.29±0.08^a^ |  |
| 183 | 1-methyl-4-prop-1-en-2-ylcyclohexa-1,3-diene | 1133 | 18368-95-1 | C_10_H_14_ | - | - | 3.46±0.33^a^ |  |
| 184 | ethylbenzene | 1127 | 100-41-4 | C_8_H_10_ | - | - | 0.3±0.1^a^ |  |
| 185 | 4,4,7-trimethyl-2,3-dihydro-1H-naphthalene | 1563 | 475-03-6 | C_13_H_18_ | - | - | 0.09±0.01^a^ |  |
| 186 | 1-methylideneindene | 1761 | 2471-84-3 | C_10_H_8_ | - | 3.86±0.82^a^ | 0.73±0.07^b^ |  |
| 187 | (4S)-1-methyl-4-prop-1-en-2-ylcyclohexene | 1197 | 5989-54-8 | C_10_H_16_ | - | - | 0.13±0.02^a^ |  |
| 188 | (3S,5R)-1,1,3,5-tetramethylcyclohexane | 1283 | 50876-32-9 | C_10_H_20_ | 0.34±0.09^a^ | - | - |  |
| **Ethers** |  |  |  |  |  |  |  |  |
| 189 | 1,1-diethoxyethane | 890 | 105-57-7 | C_6_H_14_O_2_ | 53.56±7.3^b^ | 98.35±4.82^a^ | 7.75±2.83^c^ |  |
| 190 | ethoxycyclohexane | 1044 | 932-92-3 | C_8_H_16_O | - | 2.13±0.45a | - |  |
| 191 | 2-(1,2-diethoxyethyl)furan | 1563 | 14133-54-1 | C_10_H_16_O_3_ | - | - | 0.26±0.01^a^ |  |
| 192 | 1,1-diethoxybutane | 988 | 3658-95-5 | C_8_H_18_O_2_ | - | 0.36±0.08^a^ | - |  |
| 193 | 1,1-diethoxyhexane | 1228 | 3658-93-3 | C_10_H_22_O_2_ | - | 2.01±0.54^a^ | - |  |
| 194 | 1-(1-ethoxyethoxy)-3-methylbutane | 1105 | 13442-90-5 | C_9_H_20_O_2_ | - | 0.92±0.16^a^ | - |  |
| 195 | 1-(1-ethoxyethoxy)pentane | 1096 | 13442-89-2 | C_9_H_20_O_2_ | - | - | 0.9±0.25^a^ |  |
| 196 | ethenoxyethane | 665 | 109-92-2 | C_4_H_8_O | - | 5.93±1.82^a^ | - |  |
| 197 | 2-(ethoxymethyl)furan | 1272 | 6270-56-0 | C_7_H_10_O_2_ | 0.56±0.02^b^ | 1.14±0.31^a^ | - |  |
| 198 | oxirane | 678 | 75-21-8 | C_2_H_4_O | 0.44±0.05^a^ | - | - |  |
| 199 | 1,1-diethoxy-3-methylbutane | 1063 | 3842-03-3 | C_9_H_20_O_2_ | 3.7±0.27^a^ | 6.32±2.49^a^ | - |  |
| 200 | 5-ethoxy-3-(1-ethoxyethyl)-3-methylhex-1-ene | 1215 | - | C_13_H_26_O_2_ | 7.55±0.23^a^ | 2.98±0.64^b^ | - |  |
| **Others** |  |  |  |  |  |  |  |  |
| 201 | 2-hydroxypropanamide | 1333 | 2043-43-8 | C_3_H_7_NO_2_ | 34.91±4.86^a^ | 4.95±1^b^ | 36.66±0.55^a^ |  |
| 202 | 2-pentylfuran | 1230 | 3777-69-3 | C_9_H_14_O | 1.22±0.29^b^ | 4.04±1.35^a^ | 1.1±0.31^b^ |  |
| 203 | 2,2-dimethyloxetane | 843 | 6245-99-4 | C_5_H_10_O | 2.22±0.52^a^ | - | - |  |
| 204 | 2,2-dimethyloxirane | 734 | 558-30-5 | C_4_H_8_O | 0.73±0.05^a^ | - | - |  |
| 205 | 2,4,5-trimethyl-1,3-dioxolane | 992 | 3299-32-9 | C_6_H_12_O_2_ | - | - | 34.42±0.92^a^ |  |
| 206 | 2-heptyl-1,3-dioxepane | 1055 | - | C_12_H_24_O_2_ | - | - | 0.37±0.19^a^ |  |
| 207 | 4,5-dimethyl-2-pentadecyl-1,3-dioxolane | 1268 | 56599-61-2 | C_20_H_40_O_2_ | - | - | 0.64±0^a^ |  |
| 208 | 2,4,6-trimethyl-1,3,5-trioxane | 1067 | 123-63-7 | C_6_H_12_O_3_ | 1.95±0.51^a^ | - | - |  |
| 209 | N-benzyl-N-(1-phenylethyl)nitrous amide | 1833 | - | C_15_H_16_N_2_O | - | - | 49.18±3.84^a^ |  |
| 210 | 2-ethyl-1,6-dioxaspiro[4.4]nonane | 1348 | 38401-84-2 | C_9_H_16_O_2_ | - | - | 0.15±0.02^a^ |  |
| 211 | pentan-2-yl pentyl sulfite | 1852 | - | C_10_H_24_O_3_S | - | - | 3.82±0.24^a^ |  |
| 212 | 2,3,3,4,7-pentamethyl-2H-1-benzofuran | 1705 | - | C_13_H_18_O | - | - | 0.79±0.05^a^ |  |
| 213 | 4-ethyl-2-methoxyphenol | 2030 | 2785-89-9 | C_9_H_12_O_2_ | 2.69±0.5^b^ | 9.35±3.33^a^ | - |  |
| 214 | 2,4-ditert-butylphenol | 2319 | 96-76-4 | C_14_H_22_O | 15.88±4.31^b^ | 55.99±5.46^a^ | 11.66±1.9^b^ |  |
| 215 | 4-ethylphenol | 2185 | 123-07-9 | C_8_H_10_O | - | 3.38±1.02^a^ | 0.34±0.03^b^ |  |

Notes: Data are means ± standard deviation. Different letters (a-c) within each row are significantly different (p < 0.05). " – ", not detected.
